# Sequence heterogeneity in pneumonia virus of mice reveals G gene-dependent modulation of virulence

**DOI:** 10.1128/jvi.00103-26

**Published:** 2026-06-24

**Authors:** Akinbami R. Adenugba, Patrick Bohn, Jiangyan Yu, Markus Fehrholz, Anke K. Bergmann, Redmond P. Smyth, Christine D. Krempl

**Affiliations:** 1 Institute for Virology and Immunobiology, Julius-Maximilians-Universität 9190, Würzburg, Germany; 2 Helmholtz Institute for RNA-based Infection Research, Helmholtz Centre for Infection Research, Würzburg, Germany; 3 Institute of Clinical Genetics and Genomic Medicine, University Hospital Würzburg 676759, Würzburg, Germany; 4 University Children’s Hospital, University Hospital Würzburg 676759, Würzburg, Germany; 5 Université de Strasbourg, CNRS, Architecture et Réactivité de l’ARN 27083, Strasbourg, France; Loyola University Chicago - Health Sciences Campus, Maywood, Illinois, USA

**Keywords:** pneumonia virus of mice strain J3666, attachment protein G gene, sequence polymorphism, reverse genetics, virulence

## Abstract

Pneumonia virus of mice (PVM), the mouse homolog to respiratory syncytial virus (RSV), is increasingly used as a surrogate model to study pneumovirus pathogenesis in a more natural pathogen-host relationship. Two major strains of PVM, strain 15 and J3666, are currently used in laboratories, with preferences for either one or the other based on the well-documented isolation history of strain 15, or the suggested higher virulence of strain J3666. Using conventional and long-read sequencing, we found that the PVM strain J3666 represents two distinct virus populations, which are defined by the sequence and structure of the G and SH genes encoding the putative attachment and small hydrophobic proteins, in addition to further nucleotide polymorphisms. Specifically, a nucleotide polymorphism at position 65 in the G gene results in either an upstream open reading frame (uORF) preceding the main ORF in frame, or an extension of the major G ORF by 18 codons. The impact of the different forms of the J3666-G genes on PVM was examined by generating recombinant PVMs differing exclusively in the distinctive 5′ portion of the respective G gene. This revealed that the population with an extended main G ORF was more virulent than the population with a G gene containing an uORF or the parental virus. The presence of a uORF was associated with decreased expression levels of G, whereas the virus with the extended G ORF appeared to express slightly increased levels of G, which suggests that expression levels of G may modulate virulence.

**IMPORTANCE:**

The pneumonia virus of mice strain J3666 is considered a more virulent and more suitable model for severe lower respiratory tract infections. The organization of the gene for the attachment protein G is reported to contain a small upstream open reading frame (uORF) preceding the main G ORF in frame. The translated G protein is predicted to comprise 396 amino acids. We report that this virus strain may be a mixture of two different populations, each with differing virulence. The more virulent population encodes a G protein of potentially 414 amino acids instead of a small uORF. The usage of the first start codon in this G gene organization remains to be determined. Importantly, this organization of the G gene is in line with that of several newly identified pneumoviruses, i.e., canine and swine pneumoviruses. These viruses may comprise a distinct group within the Pneumoviridae family.

## INTRODUCTION

Pneumonia virus of mice (PVM), with the official taxonomic name *murine orthopneumovirus*, is the first identified member of the genus *Orthopneumovirus* within the family *Pneumoviridae* (1, 2). An increasing number of laboratories are using the virus as a convenient experimental model to study acute respiratory disease caused by the respiratory syncytial virus (RSV) within a natural host (3). Two strains, 15 and J3666, have been characterized and are currently used in laboratories. A third independent strain, named strain Y, has been described in the literature (3, 4). In addition, murine orthopneumovirus-like sequences were identified in bats or during an approach to characterize the virome of game animals in China (5, 6). Further orthopneumoviruses, that is, canine, feline, and porcine isolates (CnPV, FePV, and SOPV), appear to be more closely related to the murine orthopneumoviruses than to RSV (7–9).

The PVM strain 15 is one of the original isolates described by Horsfall and Hahn (2). Its history is thoroughly documented in the scientific literature, thereby establishing it as a reference strain. An avirulent variant—also designated PVM 15/Warwick, resulting from continuous passage in tissue culture and plaque purification—exists in parallel to the original virulent isolate (3, 10, 11). In contrast, the isolation history of PVM strain J3666 is somewhat nebulous. The virus was first mentioned in 1995 by Easton and colleagues (10) and has subsequently been reported to originate from the same laboratory as strain 15, eventually even from the same isolate (3). The virus is reported to have a passage history predominantly in mice, although amplification of virus stocks also involved propagation in tissue culture (10, 12, 13). As a result of its passage history, the latter one has been suggested to be more virulent and, for this reason, is preferentially used by several laboratories (3).

The sequences of the complete genomes of PVM strain 15, both the virulent and attenuated variants, and strain J3666 have been described (12, 14). The single-stranded negative-sense RNA genomes contain 10 genes that potentially encode 12 proteins, in contrast to 11 proteins encoded by the RS viruses. In addition to the main open reading frame (ORF), the PVM-P mRNA encodes a second internal ORF that codes for a potential protein consisting of 137 amino acids. This protein lacks a counterpart in the RS viruses, whereas the other 11 PVM-encoded proteins appear to be functional homologs to the RSV proteins (15–18).

The pneumoviruses encode three transmembrane surface glycoproteins, namely the small hydrophobic viroporin SH, the fusion protein F, and the attachment protein G (19). The G proteins of pneumoviruses are type II transmembrane proteins, characterized by an N-terminus located within the virus particle. The ectodomain, which includes the C-terminus, comprises two-thirds of the polypeptide and is highly O-glycosylated, similarly to mucins. This overall structure, that is, the percentage of potential O-glycosylation sites, is conserved. For RSV, the nucleotide and amino acid sequences are highly variable, such that, for example, subgroups and genotypes are defined by the sequence of G. In a similar vein, the most notable differences between PVM variants and strains, respectively, appear to reside in the organization of the G gene. The G gene of the reference strain 15 is a 1,333-nucleotide sequence that encodes a G protein of 396 amino acids. This G protein is considerably larger than its RSV homolog, which has 282–319 amino acids, depending on the isolate (14). The main G ORF contains two potential in-frame start codons at positions 83 and 182, respectively, preceded by a non-translated region of 72 nucleotides. In the case of PVM strain J3666, the 5′ non-translated region appears to be divided by a small upstream open reading frame (uORF) starting with nucleotide 29 (AUG29) and coding for a potential peptide of 12 amino acids (10, 12). The start codon and uORF would be in-frame with the main G-ORF. A second form of the PVM J3666-G gene, which potentially coexists within the same virus stock, has been described. This form is marked by a nucleotide substitution that removes the stop codon of this uORF. Consequently, the substitution results in an extension of the main ORF by 54 nucleotides, which now encodes a potential G protein of 414 amino acids (11).

The function of the pneumovirus-G protein as an attachment protein is best characterized in the case of RSV. It is thought to mediate the initial binding to the target cells, that is, ciliated cells, in the airway epithelium via interaction with the chemokine receptor CX3CR1. This primary interaction appears to be essential *in vivo* but not for infection of immortalized cell lines. It has been spontaneously deleted during propagation in cell lines or by directed genetic manipulation without major effect on replication in tissue culture with some dependence on the cell line (20–22). However, the replication of these mutants was found to be significantly attenuated in mice, and a vaccine candidate containing a G deletion proved to be overattenuated in children (20, 22). These findings point to an important role as a virulence factor *in vivo*. These observations are replicated by corresponding PVM mutants, thus indicating that the PVM G protein has an *in vivo* function analogous to that of the RSV-G protein (18).

The present study revisits the potential coexistence of two G gene forms within the same PVM J3666 stock and identifies this strain as a mixture of two distinct populations. To analyze how the various G gene forms contribute to pathogenicity, the G gene of recombinant PVM 15 (rPVM) was replaced with either form described for PVM J3666. The presence of the uORF in the G gene correlated with reduced G protein expression. In contrast, the virus encoding a potentially extended G protein appeared to express slightly higher levels of the G protein. Furthermore, extending the major G ORF led to an increase in the virulence of rPVM. Thus, the heightened virulence of strain J3666, as stated in the literature, may be attributed to the population with a G gene that contains a larger G ORF.

## RESULTS

### PVM J3666 is a mixture of two distinct G gene populations

We previously reported a potential nucleotide variation at position 65 of the PVM strain J3666-G gene that would either introduce a stop codon (nucleotide 65A, negative-sense RNA), so that the G gene would contain an in-frame small uORF preceding the main G ORF, as was originally described for PVM J3666, or code for lysine (nucleotide 65U, negative-sense RNA) leading to a 54-nucleotide extension (18 codons) of the main G ORF (10, 11). Considering the elapsed time and additional passages of the PVM stock, we decided first to reconfirm our previous observation in our present PVM J3666 stock.

**Fig 1:**
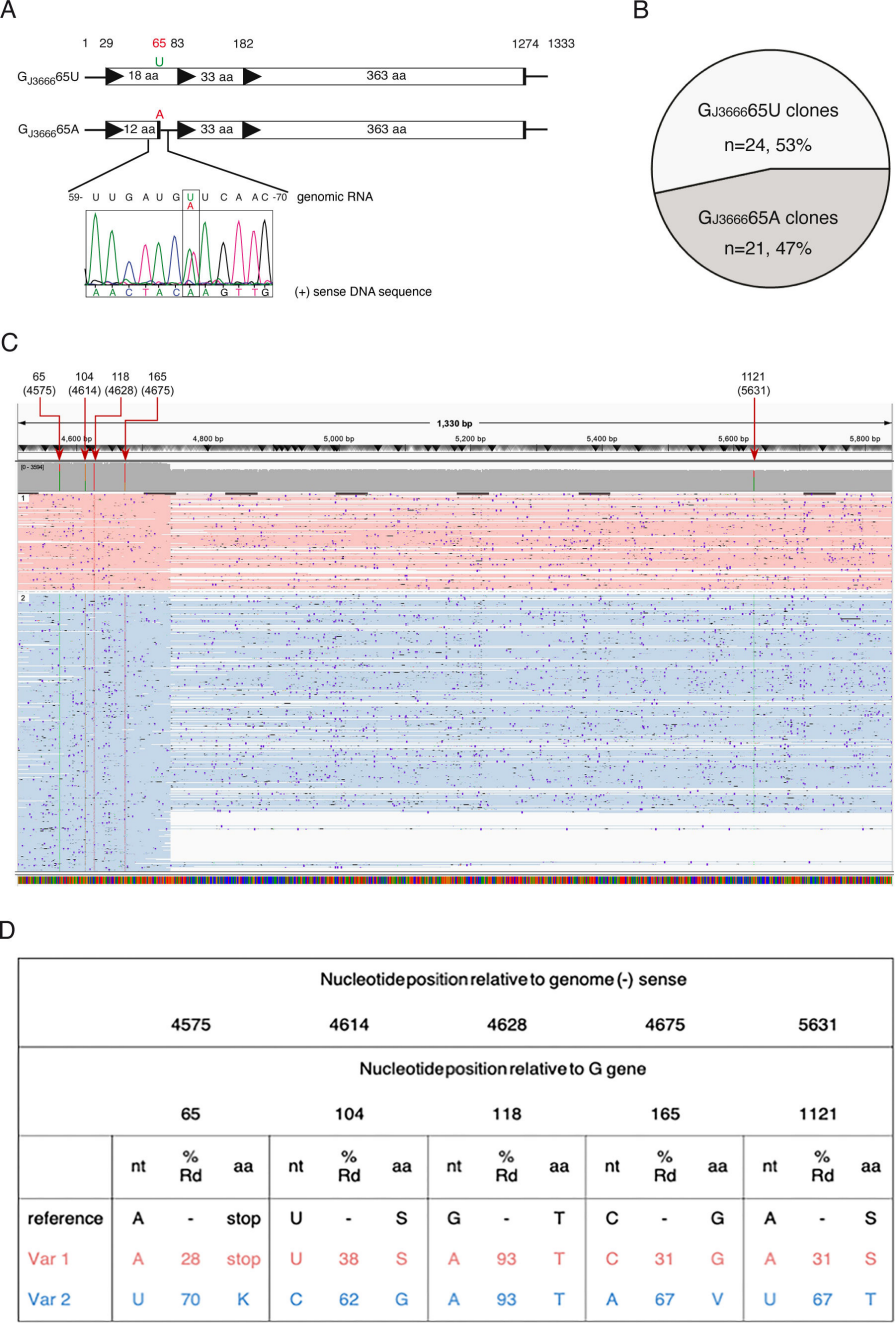
Analysis of the nucleotide 65 polymorphism of the PVM strain J3666-G gene. Total cellular RNA from PVM J3666-infected BHK-21 cells was subjected to RT-PCR amplifying a PVM fragment of 2,517 bp that covered 406 nt of the M gene, the entire SH and G genes, and 314 nt in the F gene. The consensus sequence was determined by direct sequencing of the amplification product. (**A**) Schematic representation of the G gene and the nucleotide polymorphism at position 65 with consequences on ORFs. ORFs are depicted as open rectangles with potential start codons indicated by filled triangles and stop codons by a bar. The first and last nucleotides of the gene, and the first nucleotides of the translational start and stop codons are indicated. Below the drawing, a representative electropherogram of the sequencing reaction for position 59–70 of the G gene is shown. Note that sequence data are in positive sense, whereas the genomic (−) sense RNA sequence is shown above. The overlapping peaks for nt 65 representing the first nt of the codon (underlined) for translational stop (UAG) and lysine (AAG), respectively, are boxed. (**B**) Percentage distribution of the sequence variation at position 65 within the PVM J3666 preparation. To analyze the percentage composition of the population, the RT-PCR fragments were cloned, and the G gene sequences of 45 individual clones were determined. (**C**) Confirmation of sequence variation by ONT sequencing. The haplotagged reads spanning the full G gene (position 4511–5843 of the reference genome AY753909) are visualized using the Integrative Genomics Viewer (IGV). Reads corresponding to Var1 and Var2 are shown in pink and blue, respectively. Nucleotide polymorphisms relative to the gene and genome (in brackets) are pointed out by arrows. (**D**) Percentage distribution of nucleotide variations and amino acid exchanges according to read-based phasing.

To this end, a fragment covering part of the M gene, the complete SH and G genes, and part of the F gene was amplified from total cellular RNA of infected cells by RT-PCR using a proofreading polymerase. Direct sequencing of the G gene within the fragment confirmed our previous findings regarding nucleotide variations at position 65 (A or U in negative-sense RNA) (Fig. 1A). Moreover, all other previously observed nucleotide substitutions were confirmed: nt 104 (4614 in the genome) C for U (Gly for Ser), 165 (4675 in the genome) A for C (Val for Gly), and 1121 (5631 in the genome) U for A (Thr for Ser). However, we noted underlying peaks similar to those observed for position 65, indicating nucleotide variations also for these positions (data not shown).

To determine the percentage distribution of these nucleotide variations, the described RT-PCR fragments were inserted into plasmids and sequenced. Overall, six distinct cDNA preparations were generated from three independent RNA purifications, and the cloned PCR fragments were obtained from these six preparations. Of the 45 clones analyzed, 24 clones (53%) contained a G gene with a single extended G ORF starting at AUG29 (nt 65 U, negative-sense RNA), whereas 21 clones (47%) contained a G gene encoding a small uORF (nt 65 A, negative-sense) preceding the main G ORF that starts at AUG83 (Fig. 1B). Analysis of the clones with respect to other variable nucleotide positions revealed that clones encoding an A at G gene position 65 would encode a U at position 104 (Table S1). In addition, 17 of these clones were analyzed up to position 165, where C was encoded in all cases. In comparison, the clones that encoded U in position 65, with a single exception, all encoded C in position 104, and 20 out of 23 analyzed up to position 165 encoded U in this position.

Long-read sequencing methods, such as Oxford Nanopore sequencing (Oxford Nanopore Technology, ONT), enable direct characterization of virus populations by sequencing entire viral genomes in single reads. To confirm the results obtained by conventional Sanger sequencing, we applied ONT sequencing to the virus stock used for previous experiments. We designed a sequencing strategy that enabled us to phase single nucleotide polymorphisms across the whole PVM genome. Specifically, we designed three PCRs, two of which overlapped the SH-G region. We selected the highly processive reverse-transcriptase MarathonRT and Primestar GXL polymerase for cDNA synthesis and amplification and performed long-read DNA sequencing with Kit 14 on a Flongle and MinIon R10.4.1 flow cell.

Overall, 16,024 reads (49 megabases) were generated, which matched the expected amplicon sizes and could be aligned to the reference sequence NC_006579 of PVM strain J3666. The mean sequencing depth of the G gene was 2,831×. The data confirmed the polymorphism at position 65 of the G gene (i.e., 4575 of the genome), with 28% of reads indicating A and 70% of reads indicating U (Fig. 1C and D). Furthermore, differences in position 104 (4614 of the genome), 165 (4675 of the genome), and 1121 (5631 of the genome) when coding for U in position 65 were confirmed (Fig. 1C and D). This almost homogeneous distribution of nucleotide polymorphisms pointed to two discrete populations that we preliminarily termed G_J3666_65A and G_J3666_65U based on the originally described variation.

**Fig 2:**
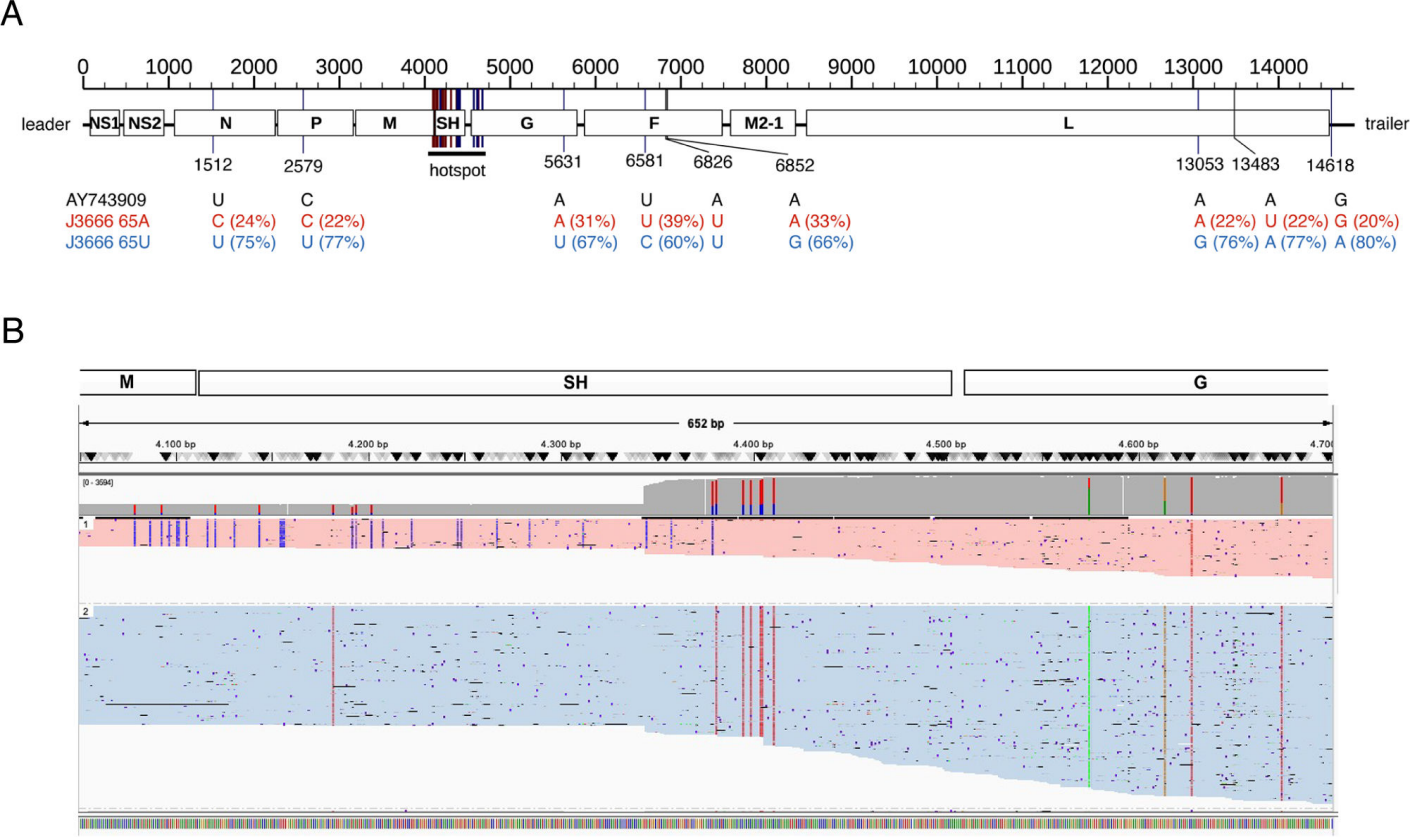
Nucleotide polymorphisms distributed over the full genome divide PVM J3666 into two discrete populations. (**A**) Schematic representation of the PVM J3666 genome with positions of polymorphisms, including the hotspot covering the 5′ noncoding part of the M gene, the entire SH gene, and the first 200 nts of the G gene. Except for the hotspot, the nucleotides in the reference sequence, as well as the polymorphisms aligning with the genomic 65A or 65U nts, are indicated below the scheme along with the percentage distribution of reads. (**B**) IGV visualization showing the accumulation of nucleotide polymorphisms in the region from nt 4052 to 4700 of the PVM J3666 genome, in the two discrete populations. The colors of the reads correspond to those presented in Fig. 1C and D. The respective genes are indicated above the ruler.

Analysis of the full genomic sequence revealed further nucleotide polymorphisms compared to the published sequence in seven out of ten genes (Fig. 2A). The 5′ non-coding region of the M gene, the SH gene, and the first 200 nts of the G gene presented themselves as a hotspot of nucleotide polymorphism, as summarized in Fig. 2B and Table S2. Overall, 34 nucleotide polymorphisms distributed throughout the M and SH genes, in addition to the ones already described for G, were identified. Most of these nucleotide polymorphisms were specific to either the G_J3666_65U or G_J3666_65A genomes, thus confirming the presence of two defined populations within PVM strain J3666 (Fig. 2B). Of note, 27 of the nucleotide exchanges involve an A to G (− sense) replacement from the reference sequence to the J3666-65A population, which is the most related sequence, but not to J3666-65U. This could point to a single hypermutation event as mediated by the adenosine deaminase acting on RNA (ADAR) 1 on vRNA (A−I, i.e., U−C on cRNA). Three of these putative hypermutations are in the M gene end signal, and two are in the SH gene start signal, respectively, which potentially affects their functionality (Table S2). Furthermore, 16 nucleotide exchanges in the SH ORF also cause amino acid substitutions.

Of the seven remaining nucleotide substitutions that represent G–A (– sense) changes from the reference sequence to the J3666-65U population, the most prominent effect would result in the expression of differentially sized SH proteins, with the A (– sense) at position 287 of the SH gene (nt 4399 of the genome) representing the first nucleotide of the stop codon, thus ending the SH ORF after 92 codons, as opposed to G at this position, which would induce a codon for glutamine.

Taking our results together, we conclude that PVM J3666 represents a mixture of two distinct virus populations, which are mainly represented by the sequence, organization, and ORF sizes of the G and SH genes.

### Recombinant PVM differing exclusively in the organization of the G genes

**Fig 3:**
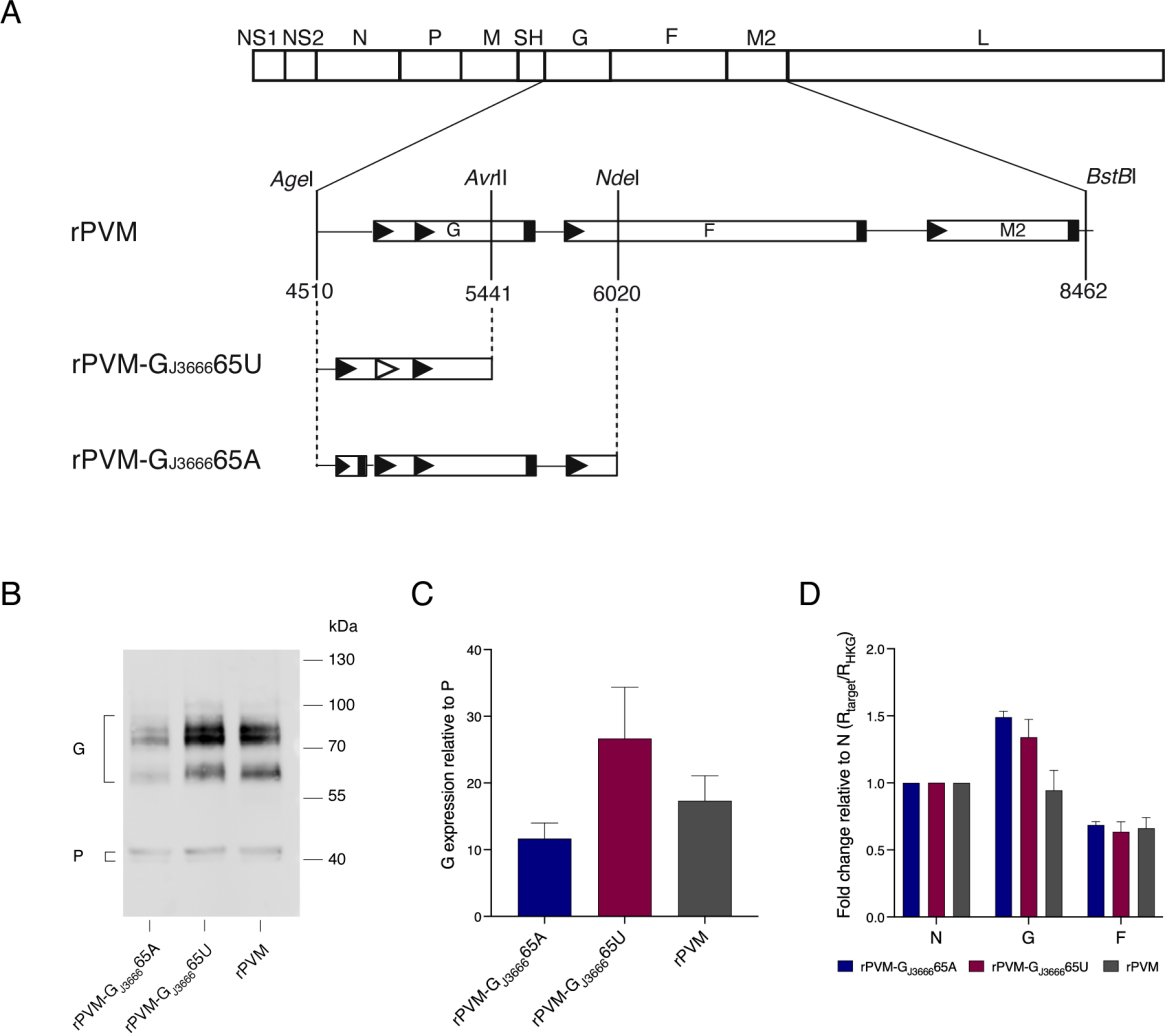
Recombinant PVM with G genes corresponding to J3666 65A or 65U, respectively. (**A**) Schematic representation of rPVM encoding the two G gene variants of strain J3666. All changes were performed using a plasmid containing an antigenomic cDNA fragment encoding the G, F, and M2 genes, which was flanked by unique *Age*I and *BstB*I restriction enzyme sites for reassembly of the antigenomic full-length plasmid. Fragments of J3666-G containing the 65U or 65A nts, as well as other variant-determining nts, were amplified from plasmids using a forward primer that added an *Age*I restriction site specific for rPVM. The original G strain 15 fragments, as indicated in the drawing, were exchanged for corresponding G_J3666_65U and G_J3666_65A fragments. Nucleotide sequences between positions 4510 and 6020 of the genomic sequence were confirmed by sequencing. Nucleotide positions of restriction enzyme sites are given relative to the rPVM genome. (B–D) G protein expression by the rPVM-G gene variants. (**B**) The expression of G protein was determined in cell lysates collected from BHK-21 cells infected with rPVM, rPVM-G_J3666_65A, or rPVM-G_J3666_65U, respectively, by western blotting. PVM proteins were detected by immunostaining using serum from a PVM-infected convalescent mouse, combined with chemiluminescence imaging using the LAS 3000 CCD camera (Fujifilm). (**C**) Chemiluminescence signals from two independent experiments were quantified using the AIDA software (v.3.20.116, Raytest, Berlin). For each lane, the amount of G isoforms per lane was expressed as the ratio of P. The standard error of the mean between runs is represented by error bars. (**D**) The transcription rate of the G, F, and N genes was determined by SYBR green-based qRT-PCR. Total cellular RNA was isolated from BHK-21 cells infected as described above, reverse-transcribed with random hexamers, and subjected to qPCR with primers specific for the indicated mRNAs. The data were analyzed with the LinRegPCR program (23). The result for each target gene was normalized to 18S rRNA and expressed as fold change relative to the amount of N mRNA. Error bars represent the standard error of the mean between two independent runs, each performed in triplicate.

Since major differences in the pathogenicity of PVM were previously attributed to the G protein (11, 18), we focused on the variability of the PVM J3666-G gene. To investigate the functional differences between the two gene variants, recombinant PVM (rPVM) based on the PVM 15 backbone were generated that would encode the G gene containing a uORF (rPVM-G_J3666_65A) or encode a potentially extended G protein (rPVM-G_J3666_65U), respectively. For the generation of rPVM-G_J3666_65U, an *Age*I site that is unique for rPVM was added upstream of the G_J3666_65U gene by PCR amplification of an appropriate G clone described above. Subsequently, a segment of the strain 15-G gene, encompassing nt 1–925, was substituted with the previously amplified fragment of G_J3666_65U (Fig. 3A). Thus, the resulting chimeric G gene also contained the nucleotide substitutions at positions 104, 165, and 1121, which are specific for the G_J3666_65U population, since the latter nucleotide is identical to that of strain 15. The generation of rPVM-G_J3666_65A involved the addition of an *Age*I site to G_J3666_65A, similar to the previously described method. In this instance, a segment of PVM 15 containing the complete G gene and 155 nucleotides of the adjacent F gene was replaced with a PCR fragment of the J3666-65A subpopulation. Notably, there are no sequence differences in the G–F intergenic region or the first 155 nucleotides of the F gene between strains 15 and J3666. Thus, despite the use of diverse cloning strategies, the resulting rPVM mutants differed exclusively in their G genes, with the complete respective G gene equaling that of the J3666 subpopulations. The parental rPVM strain 15 has been described previously and was used for comparison (18).

To characterize the recovered rPVM-G variants, we examined the expression of the G proteins. BHK-21 cells were infected with rPVM-G_J3666_65A, rPVM-G_J3666_65U, or parental rPVM, and G protein expression in cell lysates was analyzed by western blotting. The amount of G protein was quantified relative to the expression of P protein by chemiluminescence imaging. To exclude any transcriptional effects on G protein expression, mRNA levels of G, an upstream gene (N), and a downstream gene (F) were determined by quantitative RT-PCR. The results were normalized to the amount of 18S rRNA and analyzed relative to the intrinsic N mRNA levels for each virus.

This analysis revealed that the presence of the uORF moderately reduced the expression of the G protein from the G_J3666_65A gene, compared to the relative amount of G protein expressed in cells infected with rPVM or rPVM-G_J3666_65U (Fig. 3B and C). In contrast, the amount of G protein in lysates of BHK-21 cells infected with the rPVM-G_J3666_65U variant seemed to increase when compared to the amount of G protein in rPVM-infected cells. These differences in protein expression were not due to differing transcription levels, as the ratio of G/N mRNA was comparable, ranging from 0.9-fold to 1.5-fold, although it was slightly more variable than the F/N mRNA ratio (Fig. 3D).

An increase in the size of the G_J3666_65U gene-derived protein was not observed, although 18 amino acids corresponding to 2.1 kD may have been acquired due to the extension of the main ORF. This is not unexpected, since a truncation of the cytoplasmic tail for 33 amino acids could not be detected by electrophoretic mobility in SDS-PAGE (18). It is likely that the size differences would be obscured by the extensive glycosylation of the G protein. In summary, the presence of an uORF not unexpectedly negatively affects the G gene expression, whereas deleting the uORF stop codon, which extends the main ORF to AUG29 in G_J3666_65U, may enhance the expression of the G protein.

### Replication kinetics of G gene variants in cell lines

To determine the effect of the different G gene and ORF variants on replication in susceptible cell lines, multi-cycle replication kinetics were carried out using BHK-21 and macrophage-like RAW 264.7 cells infected with rPVM-G_J3666_65A, rPVM-G_J3666_65U, and rPVM, respectively, at an MOI of 0.01 PFU/cell. The virus yield was determined for days 1, 3, 5, and 7, respectively.

**Fig 4:**
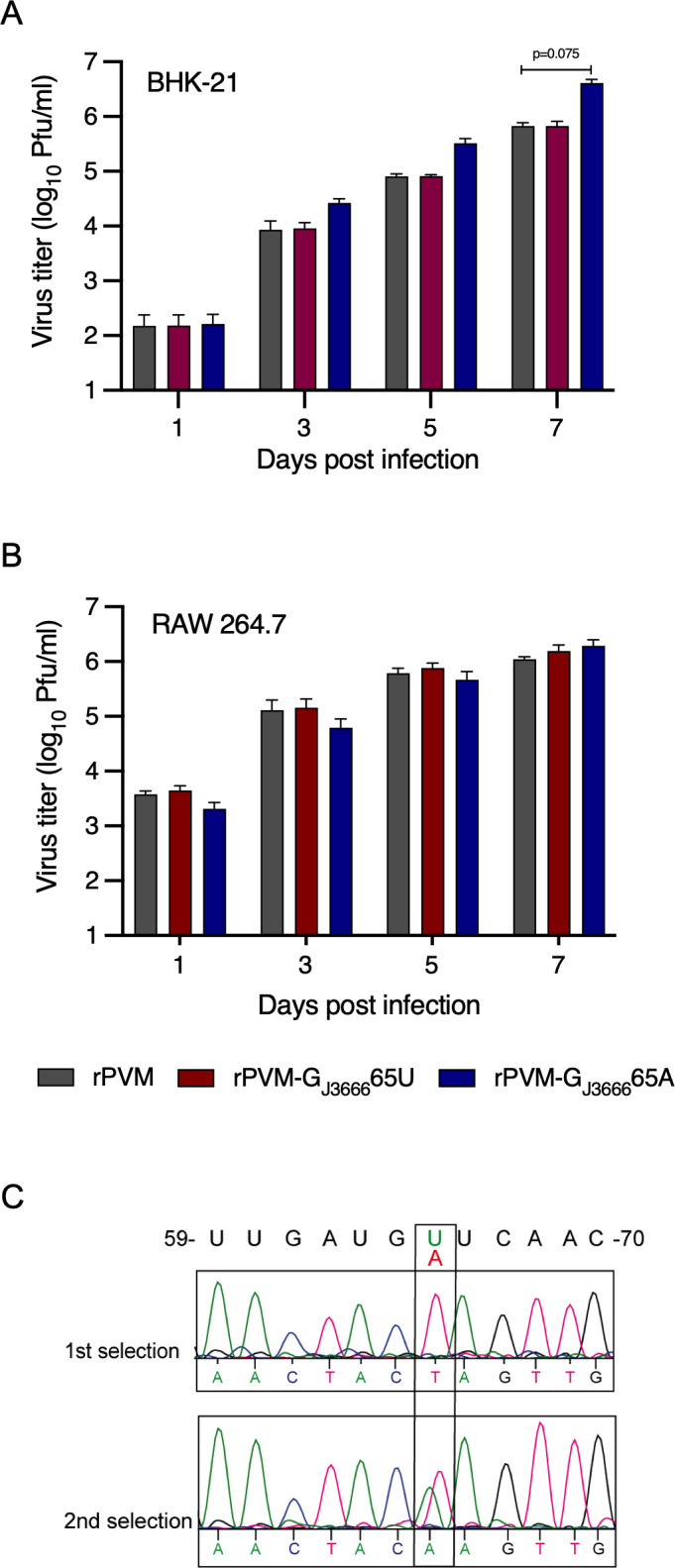
Replication kinetics of PVM-G gene variants. (**A**) BHK-21 or (**B**) RAW 264.7 cells were infected with rPVM, rPVM-G_J3666_65U, or rPVM-G_J3666_65A at an MOI of 0.01 PFU/cell. At the indicated time points, the cells and their supernatants were harvested, subjected to freeze-thaw cycles to detach cell-associated virus particles, clarified from debris, and stored for determination of virus titer by plaque assay. Data points represent the mean values of two independent experiments performed in duplicate. Standard error of the mean is represented by error bars. Statistical analysis was carried out using a mixed-effects model, followed by Tukey’s multiple comparison test. (**C**) Sequence analysis of the PVM J3666 stock following two individual *in vitro* selection experiments by sequential passaging in BHK-21 cells. Electropherograms of the sequencing reactions for nts 59–70 of the G gene after seven (first experiment) and eight passages (second experiment), respectively, are shown. Sequence data are shown in the positive-sense, whereas the genomic negative-sense RNA sequence is shown above. Peaks for nt 65 are boxed.

In BHK-21 cells, PVM with a G gene containing an extended G ORF, rPVM-G_J3666_65U, replicated almost indistinguishably from rPVM, with each virus reaching titers of approximately 7 × 10^5^ PFU/mL by day 7 (Fig. 4A). Compared to the other two viruses, rPVM-G_J3666_65A revealed a tendency to replicate more efficiently, with titers 3-fold higher by day 3 and 6-fold higher by day 7 (*P* = 0.075).

To obtain data from a second cell line susceptible to PVM, the replication kinetics were also determined using RAW 264.7 cells. In this murine macrophage-like cell line, all three PVM-G variants replicated with comparable kinetics, reaching similar titers at all time points investigated (Fig. 4B).

In order to evaluate if propagation in cell cultures may favor one of the two populations, particularly one or the other G gene variation, the mixed population was serially passaged in BHK-21 cells in two separate approaches. Following each passage, the SH and G genes were amplified from total cellular RNA of infected cells, and the region covering nucleotide 65 of the G gene was sequenced. In one experimental approach, the sequencing revealed a homogeneous G_J3666_65A population following seven passages that was confirmed by analysis of single clones, thus indicating a slight advantage of a uORF for replication in BHK-21 cells; however, in the second experiment, eight serial passages did not select for one of the two G gene populations (Fig. 4C).

This suggests that the presence of a small uORF in the G gene may confer a slight replication benefit to PVM in select cell lines. This is supported by the selection for this variant in one of two sequential passage experiments in BHK-21 cells. The extension of the G ORF does not appear to have an effect on replication efficiency.

### The organization of the G gene affects the pathogenicity in mice

Finally, to examine the effect of the varying G_J3666_-gene organization on replication and the virulence of PVM *in vivo*, BALB/c mice were infected intranasally with 150 PFU per mouse of rPVM-G_J3666_65A, rPVM-G_J3666_65U, or the parental rPVM. Disease was evaluated using weight loss as an objective assessment method. To determine replication efficiency, groups of mice were sacrificed on days 3 and 6 post-infection, respectively; the lungs were removed, and the virus load in lung homogenates was determined by plaque assay.

**Fig 5:**
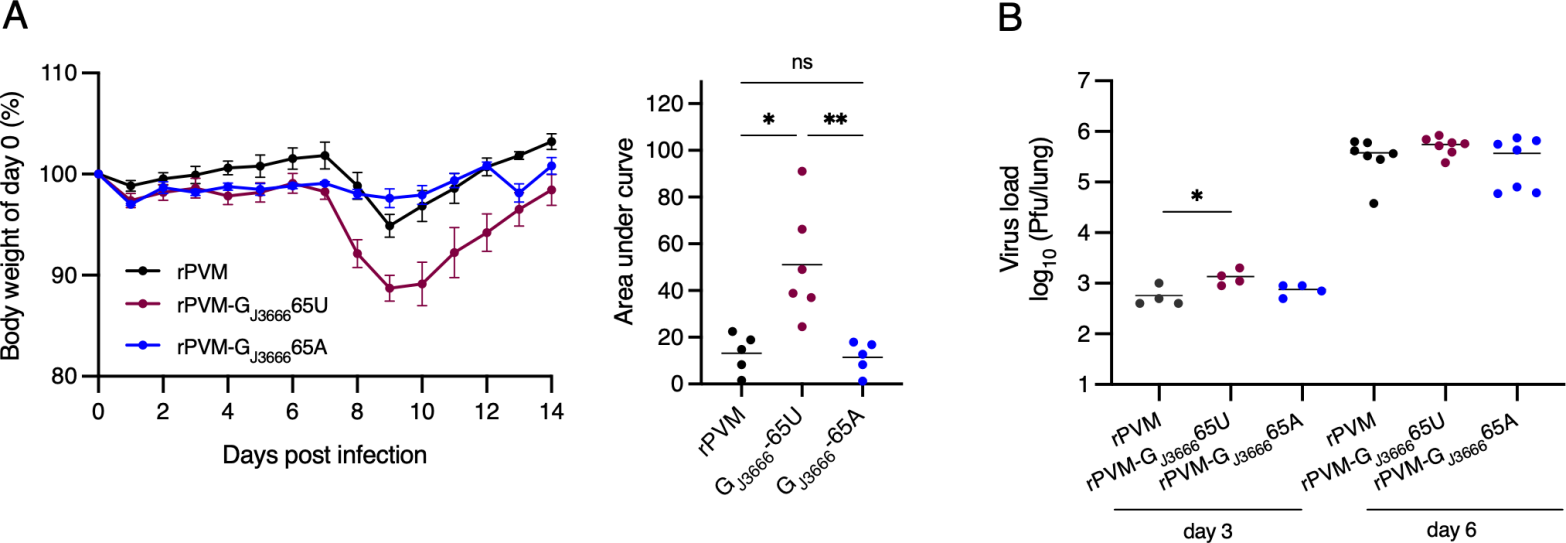
Virulence and replication of rPVM-G gene variants in the respiratory tract of BALB/c mice. Mice were infected intranasally with 150 PFU of rPVM, rPVM-G_J3666_65A, or rPVM-G_J3666_65U, respectively, in a volume of 80 µL. (**A**) For comparison of virulence, the mice were observed closely, and the body weight determined on a daily basis. Left graph: the results represent mean values of body weight relative to the weight on day 0 with standard error of the mean derived from five mice (rPVM-G_J3666_65A or rPVM) and six mice (rPVM-G_J3666_65U) per group, accumulated from 2 to 3 independent experiments. Right graph: for statistical analysis, weight development data for each animal were transformed to percent weight loss with the following formula (Y = Max(0, 100 – Y). The area under the curve between day 6 and 14 was calculated for each individual mouse, and statistical analysis was performed using the Kruskal-Wallis test, followed by Dunn’s multiple comparison test. (*) *P* < 0.05, (**) *P* < 0.01. (**B**) For the determination of replication, mice were infected as described for panel A and sacrificed at the indicated days after infection. The lungs were removed, homogenized, and the virus load was determined by plaque assay. Virus load per individual mouse is indicated by a data point, with the mean indicated by a bar. Virus loads for day 3 are derived from a single infection experiment, whereas data for day 6 were derived from 2 to 3 independent infection experiments. Statistical analysis was performed using the Kruskal-Wallis test, followed by Dunn’s multiple-comparison test (*, *P* ≤ 0.05).

Infection of mice with rPVM resulted in mild transient weight loss over 2 days, starting after day 7, with the maximum average weight loss being ca. 6% on day 9, followed by full recovery until day 12 (Fig. 5A). This weight loss was accompanied by signs of disease up to a clinical score of 2 (Fig. S1). On day 3, the average pulmonary virus load was 5.7 × 10^2^ PFU/lung, and on day 6, it was 3.8 × 10^5^ PFU/lung (Fig. 5B). In comparison, mice infected with the equivalent dose of rPVM-G_J3666_65A did not significantly lose weight or show any clinical symptoms throughout the duration of the experiment, indicating that the presence of a uORF somewhat attenuated the virulence of PVM. This attenuation appeared to be independent of replication efficiency, since pulmonary titers of rPVM-G_J3666_65A were not significantly different from those of the parental virus on both days determined (7.5 × 10^2^ PFU/lung vs. 5.7 × 10^2^ PFU/lung on day 3; 3.7 × 10^5^ PFU/lung vs. 3.8 × 10^5^ on day 6). In marked contrast, the weight loss of mice infected with rPVM-G_J3666_65U was significantly more pronounced, exceeding 10% at the peak of disease (Fig. 5A) with symptoms up to a clinical score of 3 (Fig. S1). The enhanced virulence of rPVM-G_J3666_65U was accompanied by a virus load that appeared somewhat increased, that is, 2-fold, compared to that of rPVM-infected mice at day 3, with the caveat of just four mice per group. However, the virus load leveled out at 5.5 × 10^5^ PFU/lung for rPVM-G_J3666_65A and rPVM at day 6, the peak of virus replication. Thus, the G_J3666_65U variation conferred a more virulent phenotype and may provide a slight replicative advantage *in vivo* at early time points of infection.

In summary, the two G gene variants reported for PVM J3666 have the capability to confer a differing pathogenicity phenotype to the respective virus subpopulation. The pathogenicity correlated with the G gene organization, that is, G_J3666_65U over G strain 15 and G_J3666_65A.

## DISCUSSION

Two PVM strains, 15 and J3666, are currently used as experimental models to study the pathogenesis of pneumoviruses in a natural host. Preferences of investigators for either one or the other may be based on the well-documented isolation history of strain 15 or the suggested higher virulence of strain J3666 due to frequent mouse passages (3, 10, 13). In the present study, we found that what was thought to be PVM strain J3666 actually represents a mixture of two distinct virus populations, mainly distinguished by the sequence and structure of their G and SH genes. Moreover, focusing on the G protein as a major virulence factor, we found that the two populations differ in the expression levels of the G and in their virulence.

The existence of two distinct populations was first analyzed by conventional methods that involve subcloning and sequencing of RT-PCR fragments. According to the reference sequence (NC_006579.1) (12), the J3666-G gene would encode two open reading frames, that is, the main G ORF starting at AUG83 and coding for 396 amino acids, which is preceded by a small uORF starting at AUG29 and potentially coding for a peptide of 12 amino acids. In the subpopulation described in the present study, the main G ORF could start with AUG29 and code for a polypeptide of 414 amino acids. The co-existence of two distinct PVM populations was confirmed by ONT sequencing this time covering the full genome (Fig. 1C and 2), which revealed a roughly 30%–70% distribution in favor of the population with the G ORF starting at AUG29. According to this, the population expressing a G protein of potentially 414 aa represents the major population, which corresponds to our previous data (11).

Additional population-defining features are found in the SH gene, the most characterizing one introducing a preterminal stop codon in the population potentially coding for a larger G protein. This preterminal stop codon causes shortening of the SH protein by 22 codons from 114 aa to 92 aa compared to the reference sequence. We observed overall 34 polymorphisms in the M and SH genes, five of them involving the M-gene end and the SH-gene start signals, respectively, identifying this region as a hotspot. Most of these polymorphisms were not reported in the reference sequence, are specific for the subpopulation containing a uORF in the G gene, and appear to be hypermutations that may result from a single event. When and where this event occurred is not clear. However, eight of the 27 polymorphic positions of the SH gene identified by us, that is, nucleotides 44, 70, 269, 283, 287, 292, 293, and 299, had also been reported to be variable independently from us and were accounted for by the quasispecies nature of viruses (12). This also included a size plasticity of the SH protein for 92, 96, or 114 amino acids. Of note, the origin of the isolate used in our study traces back to the research group reporting these polymorphisms. Furthermore, the polymorphism at positions 65 (U–A), 104 (U–C), 165 (C–A), and 1121 (A–U) in the G gene were already very clearly present following a single mouse passage directly after receiving this isolate (11) and, thus, were already present in the virus preparation that we had received. To our knowledge, the J3666 strain used by other laboratories traces back to the same source, eventually via interstations. Thus, we believe that this heterogeneity of strain J3666 is not likely to be specific for the virus preparation used by our lab, whereas the percentage distribution may be, depending on the propagation procedure.

In recent years, further pneumoviruses of dogs, cats, and swine have been identified that are more closely related to PVM than to the prototype orthopneumovirus human RSV (7–9, 24–32). Of note, the G genes of canine pneumoviruses (CnPV) and swine orthopneumoviruses (SOPV) are predicted to encode G proteins of 414 aa (Fig. S2) and an SH ORF of 92 aa (Fig. S3), respectively, comparable to the J3666-population identified by us. In addition, the PVM Y strain, which was isolated independently, appears to encode a G protein of potentially 414 aa (Fig. S2). This differs from what was previously annotated (GenBank accession no. JQ899033). Thus, we suggest that the PVM J3666-G-65U population represents the original form of PVM. Furthermore, we suggest that a G gene that codes for a G protein of potentially 414 aa represents the defining G gene organization for this genogroup of pneumoviruses (33). However, the usage of the different start codons and the resulting G proteins needs to be determined.

PVM J3666 has been reported to be more virulent in mice than the reference strain 15 (ATCC) (34). However, this increased virulence is attributed to the published reference sequence (NC_0065799) that would contain a uORF preceding the main ORF in the G gene. By constructing recombinant PVM containing the different G gene forms, we confirmed a heightened virulence of J3666 compared to strain 15. However, the more virulent form correlated with the G ORF starting at AUG29 and coding for a potential G protein of 414 aa, whereas the population containing a uORF preceding the main G ORF and coding for a G protein of 396 aa was the least virulent (Fig. 5). The virulence of strain 15, which encodes a G protein of 396 aa located in between, was significantly attenuated compared to the G-65U virus. Of note, nucleotide 30G in the 5′-UTR of the G-strain 15 gene (− sense) ablates a canonical start codon that otherwise would correspond to AUG29 and be the first AUG of a major G ORF.

Interestingly, the pathogenicity of the different PVM-G variants appeared to correlate with the expression levels of the G protein in infected cells, especially for the two J3666 variants. The low-pathogenic PVM with the reference sequence G gene was characterized by reduced G protein expression compared to the parental recombinant strain 15 virus, likely due to the ribosomal diverting activity of uORFs (35). Conversely, the variant with a G ORF starting at AUG29 expressed the highest levels of G protein (Fig. 3B and C). Notably, the predicted G proteins of strains 15 and J3666, which both start at AUG83, differ by only four amino acids, that is, L5F, V28G, T112A, and T347S. When the main G ORF starts at AUG29, it is in a suboptimal context for translational initiation (CAAAUGA, AUG29 underlined) and is predicted to promote ribosomal leaky scanning, compared to the improved contextual strength of AUG83 (AUCAUGG, AUG83 underlined) (36). Consequently, the majority of translation would be predicted to start with AUG83, as does the G ORF of strain 15, and polypeptides initiated at AUG29 would add up to the majority of polypeptides initiated at the downstream AUG. Thus, it is possible that increasing G protein levels rather than extending the cytoplasmic tail may cause the heightened virulence of the virus. However, further experiments are required for drawing clear conclusions, particularly regarding the usage of the three AUGs, which are outside the scope of this study.

Intriguingly, the G gene of RSV also contains a uORF that overlaps the start codon of the main ORF. This uORF has been shown to downregulate the G protein’s overall expression, but without significance for replication in infected cell lines (22). Remarkably, this uORF is conserved in all hRSV. Thus, this uORF may play a role in controlling G levels and limiting the pathogenicity of RSV.

In summary, we propose that the virulent form of PVM J3666 strains contain a G gene with an AUG29 start codon in the main ORF, as well as a 92 aa SH protein. These features appear to be typical of the most closely related pneumoviruses, such as CnPV and SOPV. Thus, we propose that these G and SH gene forms represent the natural form of PVM.

## MATERIALS AND METHODS

### Cells and viruses

The origin of PVM J3666 has been described (11) and has been passaged once in mice and three times in BHK-21 cells since receipt. Propagation of PVM J3666, PVM strain 15, and recombinant pneumonia virus of mice (rPVM), as well as determination of virus titers by plaque assay, has been described previously (18). Vero cells were cultivated in Eagle’s MEM supplemented with 10% fetal calf serum. BHK-21 cells and BSR-T7/5 cells constitutively expressing the T7 polymerase (37) were maintained in Glasgow’s MEM. RAW 264.7 cells were grown in Dulbecco’s MEM supplemented with 1× sodium pyruvate (Invitrogen) and 10% fetal calf serum.

Growth curves were performed in duplicate. BHK-21 cells and RAW 264.7 cells seeded in 12-well plates were infected at an MOI of 0.01 PFU per cell. Cells and supernatant were then collected at the indicated time points; the cell-bound virus was detached by vigorous mixing, cell debris was removed by low-speed centrifugation, and the aliquots were flash-frozen and stored at −80°C until used.

Selection experiments were performed by sequential passaging of biological PVM J3666. For each passage, BHK-21 cells were infected at an MOI of 0.01 for 7 days; then, the cells and supernatants were harvested as described above. Cell debris was used for RNA isolation, and supernatant was passaged on.

### Amplification and cloning of PVM fragments for sequence analysis

Total cellular RNA was collected from infected cells as described previously (14). The RNA was reverse-transcribed with RevertAid H minus reverse transcriptase (Thermo Scientific, Leon-Rot, Germany) and random hexamer primer (Qiagen, Hilden, Germany), followed by RNaseH treatment (NEB, Frankfurt, Germany). Thereafter, 5 µL of RT reaction was subjected to 35 cycles of PCR amplification using PVM J3666-specific primers (PVM M fw: 5′-GGTACCTTGATAGTGTGGAGCAG; PVM F rs: 5′ GGTACCAAGAATCAAACCGAGGAAC) and Phusion high-fidelity polymerase (NEB, Ipswich). Note that the primers added artificial *Acc65*I sites (underlined) to both ends of the amplicons for cloning into pGEM3zf(+) or pUC19 plasmids. Nucleotide sequencing was carried out using an ABI 3100 sequencer and the Big-Dye terminator mix version 1.1 (Applied Biosystems, Darmstadt, Germany).

### Quantification of PVM transcripts

Total cellular RNA was collected from BHK-21 cells infected at an MOI of 1 PFU/cell after 24 h with the RNeasy RNA Isolation Kit (Qiagen, Hilden, Germany) as specified by the manufacturer, and 1 µg of the RNA was reverse-transcribed as described above. The synthesized cDNAs were diluted 1:10 with H_2_O and quantified in triplicate using the iTaq universal SYBR Green supermix using the following primer pairs: GACCGACCTGATTTACCT (PVM G fw) and CACCATTGTTTAAGCCCA (PVM G rs); the primer pairs for N and F, and 18S rRNA are published (15, 38).

The specificity and sizes of the PCR products were confirmed with the melt curve and gel electrophoreses, respectively. The data were analyzed using the LingRegPCR software (23). The N, F, and G mRNA were first normalized to their respective 18S rRNA and the normalized F and G mRNA were analyzed to the normalized N mRNA.

### Nanopore sequencing of virus stock

Viral RNA of 160 µL of virus stock was extracted using the Macherey-Nagel Viral RNA mini kit. RNA was eluted in 20 µL elution buffer, followed by reverse transcription of 5 µL eluate in a 20 µL reaction containing 40 U MarathonRT in ×1 RT buffer (50 mM Tris-HCl pH 8.3, 200 mM KCl, 20% (vol/vol) glycerol) and primers RT1, PCR A re, and PCR B re. Marathon RT was purified according to Zhao et al. (39); pET-6xHis-SUMO-MarathonRT was a gift from Anna Pyle (Addgene plasmid #109029; https://n2t.net/addgene:109029; RRID:Addgene_109029). After incubation at 42°C for 2 h, the RT was inactivated by heating to 80°C for 5 min, followed by cDNA purification with the Macherey-Nagel PCR Clean-up Kit using NTC buffer and elution in 12 µL elution buffer. Next, PCRs to generate amplicons A, B, C, BC, and ABC were performed with Primestar GXL. Amplification success was confirmed on a 1% agarose gel in TAE buffer, and amplicons were purified with 1 volume of SPRI beads (Omega Biotek MagBind NGS), washed twice with 80% EtOH, and eluted in 10 µL H_2_O. Amplicons were pooled at an equimolar ratio. The pooled products were end-repaired by the addition of 2.8 µL NEB Ultra II End Repair buffer and 1.2 µL NEB Ultra II End Repair Enzyme Mix, followed by incubation at RT for 5 min and 65°C for 5 min. The end-repaired DNA was then purified and concentrated to 5 µL by SPRI beads (Omega Biotek MagBind NGS), followed by the addition of 1.5 µL of native adapter ligation barcode (SQK-NBD114.96, ONT) and 6.5 µL of Blunt/TA ligation master mix (NEB). After a 20 min incubation at RT, 1 µL EDTA (SQK-NBD114.96) was added, barcoded amplicons were pooled with other, unrelated samples containing different barcodes, and the DNA was purified with 0.4 volumes of SPRI beads, washed once with short fragment buffer (SFB, SQK-NBD114.96) and twice with 80% EtOH before elution in 30 µL H_2_O. The pooled library was then subjected to motor protein ligation by addition of 10 µL NEB 5× Quick Ligation Buffer, 5 µL Kit 14 native adapter (NA, SQK-NBD114.96), and 5 µL of high concentration T4 DNA Ligase (NEB). The ligation reaction was incubated for 20 min at RT before purification with 0.4 volumes of Ampure XP beads (SQK-NBD114.96), washed, and eluted in 15 µL elution buffer (EB, SQK-NBD114.96) at 37°C for 10 min. The resulting library was quantified with the AccuClear Ultra High Sensitivity dsDNA Quantitation Solution (Biotium, Cat. No. 31027), and 10 fmol was loaded onto a Flongle R10.4.1 flow cell (ONT, FLO-FLG114) until the flow cell reached its end of life. Data were basecalled with model r10.4.1_e8.2_400bps_sup 5 kHz, with guppy version 6.5.7. Basecalled reads were then aligned to the reference sequence NC_006579 of PVM strain J3666 using minimap2, and alignments were visualized with Integrative Genomics Viewer (IGV version 2.19.4). WhatsHap (version 2.2) based phasing analysis to separate the two populations was performed using the FA-NIVA pipeline (40). Per-position mismatch rates were extracted with perbase version 0.8.5.

### Generation of rPVM mutants encoding the G gene variants of PVM J3666

The generation of recombinant PVM strain 15 (rPVM) has been described previously (18). Exchanges of the determining parts of the G gene were performed using a plasmid containing the antigenomic cDNA fragment encoding the complete G, F, and M2 genes flanked by *Age*I and *BstB*I sites. This fragment corresponds to the fragment 3 used to assemble the original full-length cDNA clone (18). Fragments of corresponding J3666-G clones containing the 65U or 65A nucleotide, respectively, as well as other distinctive polymorphic nucleotides, were amplified using a forward primer (5-ACCGGTAGGATAATCTACCTATTG; *Age*I site is underlined) binding to the G gene start signal that would also add an *Age*I restriction site upstream of G that is unique for rPVM, and a reverse primer (5- TCCACTGCACTACTATAGATTGC) that binds 161 nucleotides downstream of the G gene in F. A fragment of the strain 15-G encompassing nt 1–931 was replaced with the equivalent portion from the G_J3666_65U amplificate using the *Age*I and a naturally occurring *Avr*II site. For replacement of the strain 15-G with G_J3666_65A, the entire G gene and the next 155 nucleotides downstream of the G gene were exchanged via *Age*I and naturally occurring *Nde*I site (Fig. 3A). The resulting GJ3666-F-M2 fragments were used to exchange the corresponding portion of the original full-length cDNA clone pPVM via *Age*I and *BstB*I to obtain pPVM-G_J3666_65U and pPVM-G_J3666_65A, respectively. Recombinant PVM-G_J3666_65U and rPVM-G_J3666_65A were then recovered as previously described (18). The sequences of replaced portions were confirmed before virus recovery and in the final virus stock after recovery as described above.

### Western blot analysis

Total protein lysates were prepared from BHK-21 cells infected at an MOI of 0.1 per cell for 4 days. Cells were washed twice and collected with a cell scraper into ice-cold phosphate-buffered saline (PBS). The cells were dissolved into 150 µL of PBS and lysed with an equal amount of 2× Laemmli buffer (41). The lysates were homogenized using QIAshredder (Qiagen), and the total protein yields were quantified using the Roti-Quant Universal Kit (Roth, Karlsruhe, Germany). Equal amounts of protein were separated on 8% SDS-containing polyacrylamide gels and transferred onto Protan blotting membrane (Whatmann GE, Freiburg, Germany). Following blocking with 5% non-fat milk powder in PBS, PVM-specific proteins were detected with polyclonal serum collected from a convalescent rPVM-infected mouse combined with a secondary goat anti-mouse HRP-conjugated antibody (Millipore, Darmstadt, Germany). Images were recorded with the LAS 3000 CCD camera (Fujifilm). Two different blots from two similar infections were quantified using the AIDA software (Raytest Straubenhardt, Germany), and the results were analyzed with GraphPad Prism 11.0.1 (GraphPad, La Jolla, USA).

### Animal experiments

Six- to 9-week-old BALB/c mice were intranasally infected with a sublethal dose (150 PFU in 80 µL of phosphate-buffered saline) of virus suspension under isoflurane anesthesia. Daily weight estimation was used to monitor the virulence of the virus. For the determination of virus titers, 4 mice per group were sacrificed on day 3 and 6–7 mice per virus on day 6 post-infection; the lungs were removed and homogenized in 3 mL of EMEM containing 10% FCS, 50 mM HEPES, and 100 mM MgSO_4_. The clarified homogenates were used for virus titration on Vero cells.

### Statistical analysis

Statistical analyses were performed with GraphPad Prism 11.0.1 using appropriate tests and an alpha-value of 0.05 to establish statistical significance, unless otherwise stated.

## Data Availability

ONT sequencing data for the PVM J3666 mixed population are available from the European Nucleotide Archive (ENA) under accession number PRJEB110830.

## SUPPLEMENTAL MATERIAL

Supplemental material jvi.00103-26-s0001.pdf Tables S1 and S2; Fig. S1 to S3.

ASM does not own the copyrights to Supplemental Material that may be linked to, or accessed through, an article. The authors have granted ASM a non-exclusive, world-wide license to publish the Supplemental Material files. Please contact the corresponding author directly for reuse.
