# Supplementary material for: Sequence heterogeneity in pneumonia virus of mice reveals G gene-dependent modulation of virulence
Source: J Virol. 2026 Jun 24;100(7):e00103-26. doi: 10.1128/jvi.00103-26 (PMC13386830; doi:10.1128/jvi.00103-26)
Supplement: Supplemental material — Tables S1 and S2; Fig. S1 to S3. [file jvi.00103-26-s0001.pdf]

Table S1: Alignment of nucleotide polymorphisms for G-J3666 clones

G variants	Nucleotide position (rel. to G gene)			
	65	104	165	1121
G_{J3666}65U	U/Lys (24/24)	C/Gly (23/24)	A/Val (20/23)	U/Thr (4/4)
		U/Ser (1/24)	C/Gly (3/23)	
G_{J3666}65A	A/Stop (21/21)	U/Ser (21/21)	C/Gly (17/17)	A/Ser (5/6)
				U/Thr (1/6)

Nucleotides corresponding to the indicated positions within the G gene variants G_{J3666}65U and G_{J3666}65A (negative-sense RNA) and the encoded amino acids are shown. The number of clones examined per location is indicated in the bracket: the numerator is the number of clones that contained the nucleobase shown in the table while the denominator is the total number of clones sampled per site of G gene variant

Table S2: Nucleotide and amino acid differences of the two variants compared to the reference sequence

			Nucleotide sequences are in positive sense							
			AY743909		Var 1 (65A)			Var 2 (65U)		
Gene	Position in genome	Position relative to nt 1 of gene	Nucleotide	Amino acid	Nucleotide	% Reads	Amino acid	Nucleotide	% Reads	Amino acid
M	4070	900	U	none	C	18	none	U	82	none
	4087	908	U	none	C	18	none	U	81	none
	4093	914	U	none	C	19	none	U	81	none
	4097	918	U	none	C	18	none	U	81	none
	4101	922	U	none[a]	C	19	none[a]	U	81	none[a]
	4102	923	U	none[a]	C	19	none[a]	U	80	none[a]
	4106	927	U	none[a]	C	18	none[a]	U	81	none[a]
SH	4117	5	U	none[b]	C	18	none[b]	U	81	none[b]
	4121	9	U	none[b]	C	18	none[b]	U	80	none[b]
	4131	17	U	Pro	C	18	Pro	U	80	Pro
	4144	32	U	Tyr	C	19	His	U	80	Tyr
	4155	43	U	Thr	C	18	Thr	U	82	Thr
	4156	44	U	Phe	C	18	Pro	U	81	Phe
	4157	45	U	Phe	C	18	Pro	U	82	Phe
	4182	70	C	Arg	C	21	Arg	U	77	Arg
	4192	80	U	Tyr	C	19	His	U	79	Tyr
	4194	82	U	Tyr	C	20	His	U	78	Tyr
	4202	90	U	Leu	C	19	Pro	U	81	Leu
	4208	96	U	Leu	C	18	Pro	U	82	Leu
	4223	111	U	Leu	C	18	Pro	U	82	Leu
	4247	135	U	Val	C	19	Ala	U	80	Val
	4249	137	U	Cys	C	19	Arg	U	81	Cys
	4267	155	U	Cys	C	17	Arg	U	82	Cys
	4284	172	U	Ile	C	18	Ile	U	81	Ile
	4312	200	U	Cys	C	18	Arg	U	80	Cys
	4345	233	U	Tyr	C	7	His	U	93	Tyr
	4358	246	U	Val	C	6	Ala	U	94	Val
	4379	267	U	Leu	C	28	Pro	U	71	Leu
	4381	269	C	His	C	31	His	U	69	Tyr
	4395	283	C	Thr	C	30	Thr	U	69	Thr
	4399	287	C	Gln	C	30	Gln	U	70	stop
	4404	292	C	His	C	30	His	U	70	none
	4405	293	C	His	C	30	His	U	70	none
	4411	299	C	His	C	29	His	U	70	none

[a]Gene end sequence
[b]Gene start sequence

The nucleotides corresponding to the indicated positions in the genome and within the M and SH genes of the reference sequence (GenBank accession number AY43909), variant 1 (G_{J3666}65A) and variant 2 (G_{J3666}65U) are shown as positive-sense RNA. Also shown are the encoded amino acids, or if the nucleotides belong to a noncoding, respective cis-acting sequences.

Supplementary Figure S1

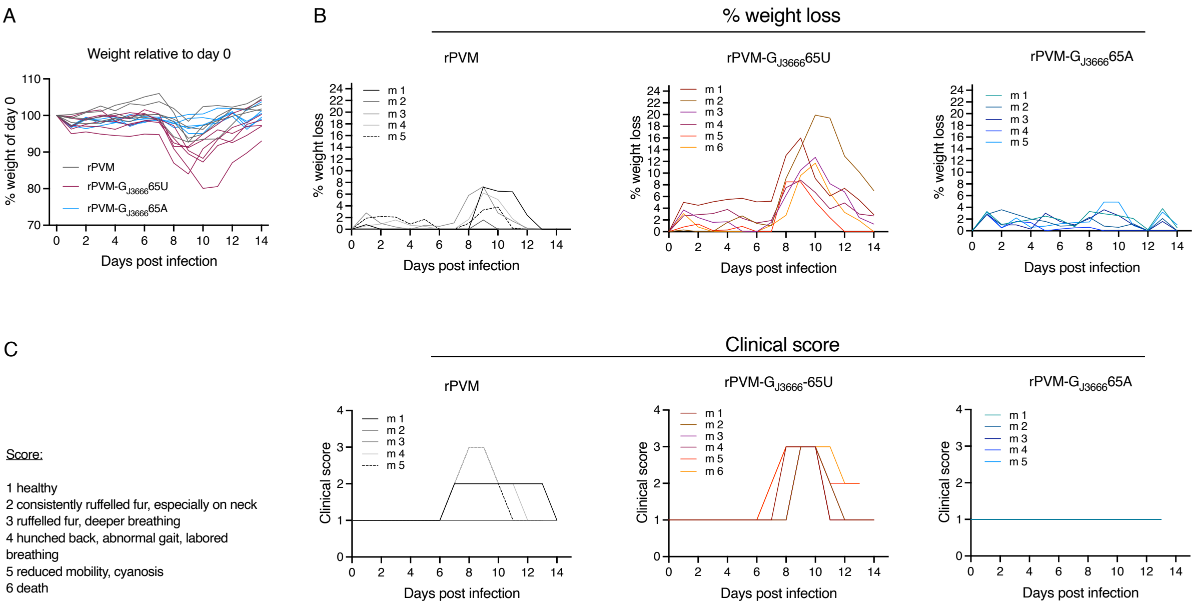

Figure S1. Virulence date for each individual mouse. (A) Weight development relative to the body weight on day 0. (B) Percent weight loss transformed with the following formula (Y=Max(0, 100 – Y). (C) Clinical scores for each animal. The score used is indicated on the left.

Supplementary Figure S2

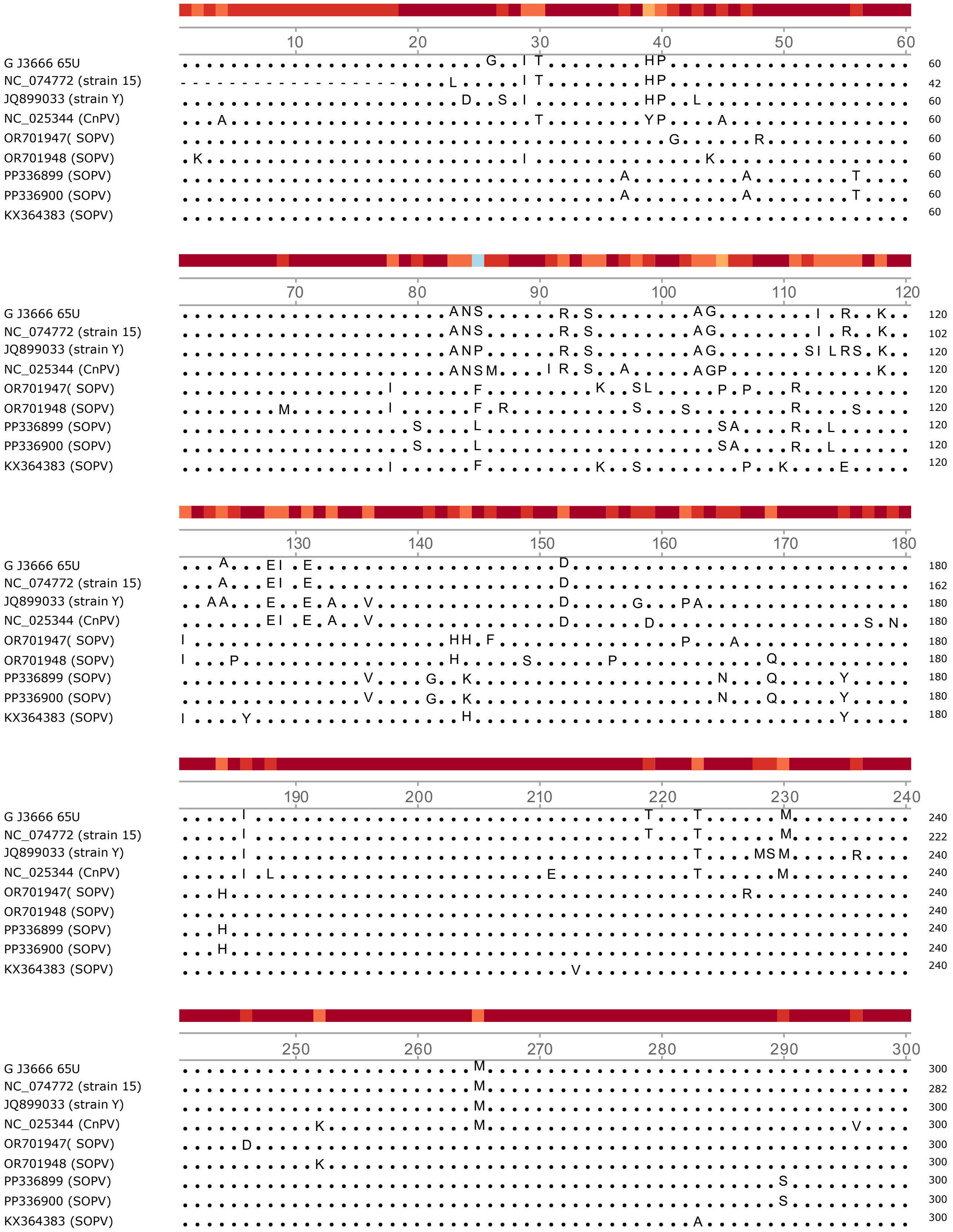

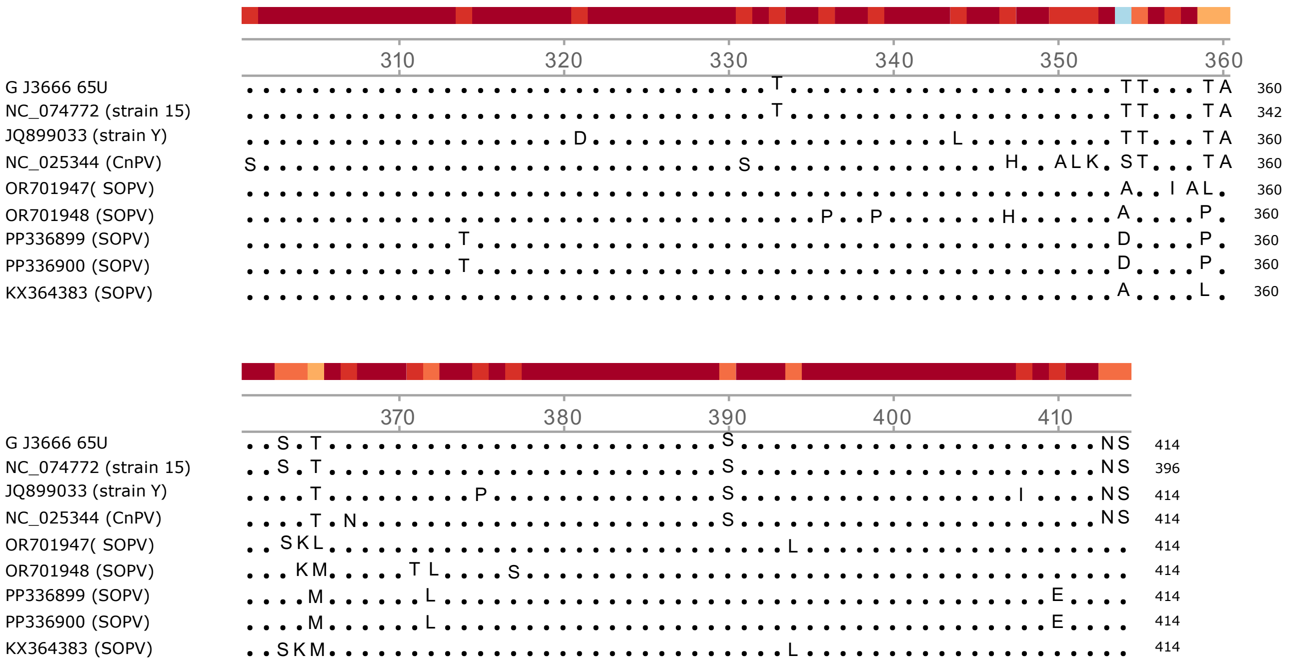

Figure S2. Alignment of PVM-related pneumovirus G proteins. Except for the first sequence which represents that of G J3666 with 414 amino acids described here, all sequences are identified by the GeneBank accession number and the species. The first three sequences belong to PVM G proteins.

Supplementary Figure S3

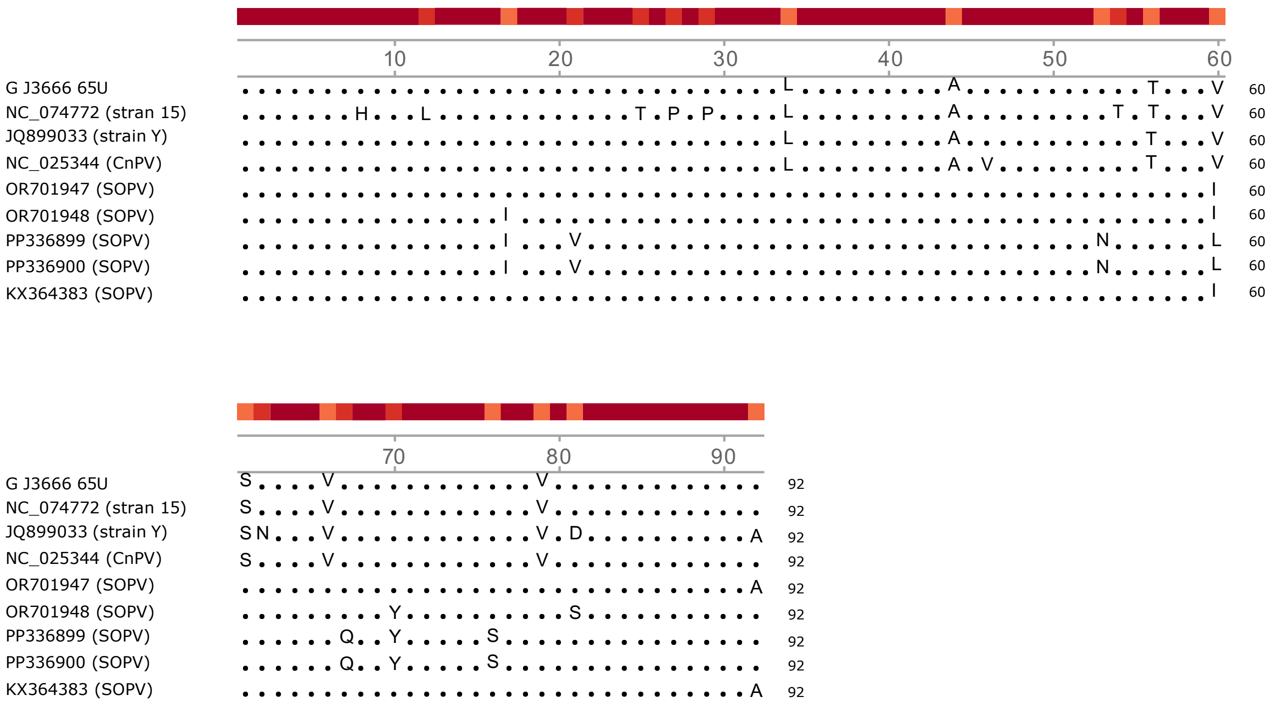

Figure S3. Alignment of PVM-related pneumovirus SH proteins. Except for the first sequence which represents that of SH J3666 with 92 amino acids described here, all sequences are identified by the GeneBank accession number and the species. The first three sequences belong to PVM SH proteins.
